# Li^+^/H^+^ exchange of Li_7_La_3_Zr_2_O_12_ single and polycrystals investigated by quantitative LIBS depth profiling

**DOI:** 10.1039/d2ma00845a

**Published:** 2022-10-24

**Authors:** Stefan Smetaczek, Andreas Limbeck, Veronika Zeller, Joseph Ring, Steffen Ganschow, Daniel Rettenwander, Jürgen Fleig

**Affiliations:** Institute of Chemical Technologies and Analytics, TU Wien Vienna Austria juergen.fleig@tuwien.ac.at andreas.limbeck@tuwien.ac.at; Leibniz-Institut für Kristallzüchtung Berlin Germany; Department of Material Science and Engineering, NTNU Norwegian University of Science and Technology Trondheim Norway; International Christian Doppler Laboratory for Solid-State Batteries, NTNU Norwegian University of Science and Technology Trondheim Norway

## Abstract

Li_7_La_3_Zr_2_O_12_ (LLZO) garnets are highly attractive to be used as solid electrolyte in solid-state Li batteries. However, LLZO suffers from chemical interaction with air and humidity, causing Li^+^/H^+^ exchange with detrimental implication on its performance, processing and scalability. To better understand the kinetics of the detrimental Li^+^/H^+^ exchange and its dependence on microstructural features, accelerated Li^+^/H^+^ exchange experiments were performed on single crystalline and polycrystalline LLZO, exposed for 80 minutes to 80 °C hot water. The resulting chemical changes were quantified by analytical methods, *i.e.* inductively coupled plasma optical emission spectroscopy (ICP-OES) and laser induced breakdown spectroscopy (LIBS). From the time dependence of the Li^+^ enrichment in the water, measured by ICP-OES, a bulk interdiffusion coefficient of Li^+^/H^+^ could be determined (7 × 10^−17^ m^2^ s^−1^ at 80 °C). Depth dependent concentrations were obtained from the LIBS data for both ions after establishing a calibration method enabling not only Li^+^ but also H^+^ quantification in the solid electrolyte. Short interdiffusion lengths in the 1 μm range are found for the single crystalline Ga:LLZO, in accordance with the measured bulk diffusion coefficient. In polycrystalline Ta:LLZO, however, very long diffusion tails in the 20 μm range and ion exchange fractions up to about 70% are observed. Those are attributed to fast ion interdiffusion along grain boundaries. The severe compositional changes also strongly affect the electrical properties measured by impedance spectroscopy. This study highlights that microstructural effects may be decisive for the Li^+^/H^+^ ion exchange kinetics of LLZO.

## Abstract 2

Pronounced Li^+^/H^+^ exchange of doped Li_7_La_3_Zr_2_O_12_ (LLZO) takes place in hot water. LIBS and ICP-OES analysis reveal the importance of grain boundaries in this ion exchange process.

## Graphical abstract

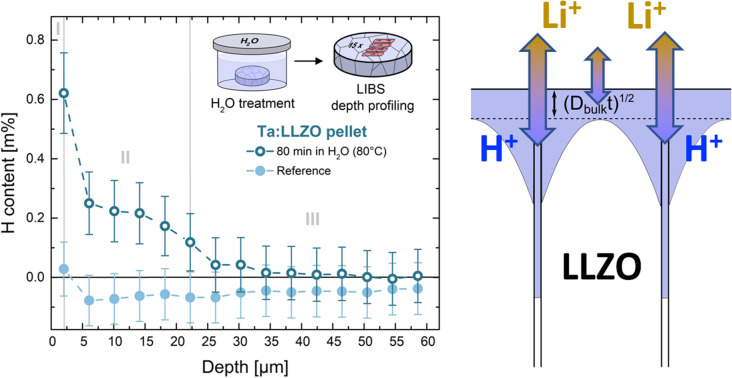

## 1 Introduction

Solid electrolytes may become key components for next-generation Li-ion batteries, featuring enhanced safety as well as increased energy densities. Among the most promising solid Li-ion conductors is the Li stuffed garnet Li_7_La_3_Zr_2_O_12_ (LLZO), which was first reported in 2007.^1^ Beside its high Li-ion conductivity (up to about 10^−3^ S cm^−1^ at ambient temperature), LLZO also exhibits chemical stability against elemental Li, enabling its use in Li–metal batteries.^1–4^ However, sensitivity in humid/aqueous environment turned out to be a critical material property of LLZO, with disadvantageous consequences for processing, interfacial stability and electrochemical performance. Several studies reported that LLZO undergoes Li^+^/H^+^ exchange (*i.e.*, Li^+^ in the garnet lattice gets replaced by H^+^) in contact with humidity.^5–7^ The process is accompanied by the formation of LiOH·H_2_O on the sample surface, which (partly) reacts to Li_2_CO_3_ when exposed to ambient air.^8,9^ A passivation layer is formed, which protects the material underneath from further degradation to some extent,^9^ but is also known to negatively affect the interfacial properties with electrodes.^10–12^ Since both LiOH and Li_2_CO_3_ are water-soluble, these secondary phases formed on the sample surface are removed in contact with water and thus do not (directly) hamper further Li^+^/H^+^ exchange in aqueous environment.^12^

Earlier studies also showed that water treatment promotes the transition from tetragonal LLZO to the highly conductive cubic polymorph.^5^ Cubic LLZO, on the other hand, seems to be stable even for high Li^+^/H^+^ exchange up to 75%.^13–16^ Despite several investigations focussing on site occupancy after Li^+^/H^+^ exchange, it is still an unsettled matter, which Li sites are preferably vacated.^13,15–18^ Recently, Redhammer *et al.*^18^ indicated that the site occupation behaviour of tantalum stabilized LLZO (Ta:LLZO), together with exchange rate, exchange capacity, and structural stability, strongly depend on the composition. Grain boundaries were shown to be more susceptible to moisture than grains.^6,19,20^ This is also in agreement with reports of an increased grain boundary resistance after the immersion of LLZO in water.^20,21^

Li^+^/H^+^ exchange ranging from 29% to 75% was reported for LLZO powders immersed in H_2_O.^7^ Although a rapid pH increase indicates high reaction rates within the first seconds,^13,16^ the exchange was shown to continue at reduced rate up to several days^22^ or even weeks.^18^ Interestingly, the Li^+^/H^+^ exchange is reversible to some extent when placing the protonated samples in strong basic Li^+^ containing solutions.^13,16^ Besides immersion time and water temperature, the rate and extent of Li^+^/H^+^ exchange depends on particle size: samples with high surface area exchange more quickly than samples consisting of large particles.^7^ This is in agreement with experiments performed on LLZO pellets by Yow *et al.*,^22^ which show an Li^+^/H^+^ exchange of only 8.8% even after one week of immersion. The authors concluded that only a thin layer close to the surface experiences considerable H^+^ incorporation and that ion diffusion inside the garnet is the rate-determining step of the ion exchange.^22^

Since Li^+^/H^+^ exchange only takes place at the surface, depth-resolved information is of major interest to gain insights in the kinetics of the process as well as to determine the true extent of the ion exchange. However, studies providing such information are scare. Brugge *et al.*^20^ conducted secondary ion mass spectrometry (SIMS) depth-profiling experiments on Ga-stabilized LLZO (Ga:LLZO) pellets immersed in a H_2_O bath at 100 °C for up to 30 min, revealing that the Li^+^/H^+^ exchanged region extends as far as 1.35 μm into the sample. The H^+^ diffusion coefficient determined in this study is in reasonable agreement with results obtained by Hiebl *et al.*,^15^ who conducted a long-term X-ray diffraction (XRD) study on an Al-stabilized LLZO (Al:LLZO) single crystal exposed to humid air.

Quantitative measurements of protons in LLZO, however, are hardly available. Among the very few analytical techniques capable of direct hydrogen detection in an oxide is laser induced breakdown spectroscopy (LIBS), which was also shown to be a powerful tool for spatially resolved cation determination of (assumably H-free) LLZO.^11,23–25^ In contrast to SIMS, which usually only provides semi-quantitative information, LIBS enables quantitative analysis of hydrogen in solids.^26–30^ Furthermore, while being inferior to SIMS in terms of spatial resolution, LIBS is less limited when it comes to sampling depth (*i.e.*, where deeper sample layers are reached) and the analysis of macroscopic areas (*i.e.*, when larger areas can be analyzed). Such a depth dependent quantitative detection of protons in LLZO is highly desirable to extend knowledge on the Li^+^/H^+^ exchange kinetics. Moreover, comparison of proton concentrations in polycrystalline and single crystalline LLZO may further reveal the importance of microstructural effects.

The Li^+^/H^+^ exchange behaviour of Ta:LLZO polycrystalline pellets as well as Ga:LLZO single crystals is therefore investigated in this study. The samples were immersed in an ultrapure water bath at 80 °C for 80 min and subsequently the chemical composition of the LLZO samples and of the water was analysed by LIBS depth profiling and inductively coupled plasma optical emission spectroscopy (ICP-OES), respectively. The experiments reveal strongly enhanced amounts of protons in the polycrystalline sample, emphasizing the importance of grain boundaries for the water sensitivity of LLZO.

## 2 Experimental

### 2.1 H_2_O exposure

All experiments presented in this study were conducted on two kinds of cubic LLZO samples:

•Ta:LLZO polycrystals with a nominal composition of Li_6.4_La_3_Zr_1.4_Ta_0.6_O_12_ purchased from Toshima Manufacturing Co., Ltd. (Japan), typical grain size of 2–4 μm, relative density of approx. 99%.

•Ga:LLZO single crystals with a composition of Li_6.43_Ga_0.14_La_2.84_Zr_2_O_11.68_ (normalized to 2 Zr per formula unit (pfu)) grown by the Czochralski method directly from the melt using previously dried high purity (99.99% or better) metal oxides or carbonates (in case of Li). More information about the sample synthesis and characterisation can be found elsewhere.^31^

In order to ensure a well-defined sample surface as well as to remove potential surface contaminations such as Li_2_CO_3_,^11^ all samples were first polished using SiC grinding paper (P4000). The samples were stored under Ar atmosphere between all experiments to minimize contact to air, which was kept below 2 min for all samples and thus should not affect the results of this study.

To investigate Li^+^/H^+^ exchange caused by exposure to H_2_O, LLZO samples were immersed in ultrapure water (18.2 MΩ cm^−1^ at room temperature) heated to about 80 °C in polyethylene testing tubes. The ultrapure water was obtained by a Barnstead™ Easypure™ II (Thermo Fisher Scientific, USA). Individual samples were immersed in about 5 ml H_2_O for a total duration of 80 min. The deionized water was replaced three times (after 5 min, 30 min, and 55 min) during the experiment. After the immersion, the samples were quenched by dipping them into H_2_O cooled down to 5 °C.

To ensure that the released cations are stabilized in the aqueous solutions (*i.e.*, to prevent adsorption at the testing tube walls), a 1/100 (v/v) mixture of hydrofluoric acid (40 m%, Emsure®, Merck, Germany) and nitric acid (65 m%, Emsure®, Merck, Germany) was added to all water fractions after the Li^+^/H^+^ exchange experiment, resulting in final nitric acid concentration of 0.65 m%. All samples were stored at 5 °C until chemical analysis as well as between all further measurements.

### 2.2 ICP-OES measurements

To investigate the release of cations, all water fractions used for sample immersion were analysed by ICP-OES. For the measurements, an iCAP 6500 RAD (Thermo Fisher Scientific, USA) equipped with an echelle-type monochromator and a charge injection device (CID) detector was used. Qtegra software provided by the manufacturer of the instrument was used for data acquisition. Introduction of the samples was performed using an ASX-520 autosampler (CETAC Technologies, USA), PTFE tubing, and a sample introduction kit consisting of a conventional Meinhard high-solids quartz nebulizer and a quartz cyclone spray chamber without ascension tube. A plasma torch containing a quartz injector tube with 1.5 mm inner diameter was used for the analysis. Detailed information about the used instrument parameters is given in Table 1.

**Table 1:** Instrumental setting ICP-OES analysis

	Thermo iCAP 6500 RAD	
RF power	1200 W	
Radial observation height	12 mm	
Plasma gas flow (Ar)	12 l min^−1^	
Nebulizer gas flow (Ar)	0.6 l min^−1^	
Auxiliary gas flow (Ar)	0.8 l min^−1^	
Integration time	5 s	
Replicates per sample	5	
Purge pump rate	1.6 ml min^−1^	
Sample flow rate	0.8 ml min^−1^	
Analytical wavelengths		
Eu (Internal standard)	381.967 nm	
Ga	294.364 nm	417.206 nm^a^
La	333.749 nm	412.323 nm^a^
Li	610.362 nm	670.784 nm^a^
Ta	240.063 nm	268.517 nm^a^
Zr	339.198 nm	343.823 nm^a^

a Used for quantification.

Signal quantification was employed *via* univariate calibration using certified single element ICP-standard solutions (Certipur®, Merck, Germany). Calibration standards containing Ga, La, Li, Ta, and Zr in concentration ranging from 1 to 1000 μg kg^−1^ were prepared by mixing the corresponding single element standards and diluting the obtained stock solution using a 1/100 (v/v) dilution of nitric acid (0.65 m%). An Eu ICP-standard solution (Certipur®, Merck, Germany) was added to all standard and sample solutions to a final concentration of 200 μg kg^−1^ and acted as internal standard for the analysis.

### 2.3 LIBS measurements

Changes in the H as well as Li content within the LLZO samples were probed by means of LIBS after the 80 minutes exposure to hot water (see above). Measurements were conducted using a commercially available J200 LIBS system (Applied Spectra Inc., USA) equipped with a 266 nm frequency quadrupled Nd:YAG laser and a six-channel Czerny–Turner type spectrometer covering a wavelength range from 188 to 1048 nm. For data collection, Axiom 2.0 software provided by the manufacturer of the instrument was employed. Detailed information about the instrumental parameters used for the analysis is shown in Table 2, which allowed simultaneous analysis of all target analysts with sufficient sensitivity.

**Table 2:** Instrumental setting LIBS analysis

LIBS instrumentation	J200
Pulse duration	5 ns
Output energy	2.3 mJ
Beam diameter	60 μm
Scan speed	0.12 mm s^−1^
Repetition rate	10 Hz
Beam geometry	Circular
Gate delay	0.1 μs
Gate width	1.05 ms
Atmosphere	He
Gas flow	2 l min^−1^

In addition to samples exposed to H_2_O, also samples not treated with hot deionized water were measured and served as reference for the analysis. To check the influence of potential H_2_O residues (*e.g.* in the sample pores), all samples including the reference ones were dipped (again) into 5 °C cold distilled water directly before transfer to the sample chamber, thus ensuring equal measurement conditions (*i.e.*, H signal from excess H_2_O is also visible in reference spectra). Before the start of the measurement, the samples were dried inside the sample chamber under He atmosphere (constant gas flow of 2 l min^−1^) for 2 h at room temperature.

For signal quantification, matrix-matched standards with variable H content (ranging from 0.00 m% to 0.64 m% nominally) were prepared by pressing different mixtures of calcinated Al:LLZO precursors and La(OH)_3_ (99.9%, Sigma-Aldrich, USA) powder into pellets. The used Al:LLZO precursor powder (nominal composition Li_7.04_Al_0.2_La_3_Zr_2_O_12_ including excess of Li precursor) was synthesized using a conventional high-temperature sintering route based on the procedure described by Wagner *et al.*^32^ For each standard, a total amount of about 1 g powder mixture was homogenized using an agate mortar, transferred into a flexible silicone rubber mould, and cold pressed using a mechanical isostatic press (Paul-Otto Weber, Germany) at a pressure of 300 MPa. In addition to in-house prepared pressed pellets, a pristine Ga:LLZO single crystal, which was assumed to be hydrogen free, was used as calibration standard. All standards were polished with SiC grinding paper (P4000) directly before transfer into the sample chamber of the LIBS instrument.

Ablation patterns consisting of one line scan with a length of 1.2 mm were employed for all measurements. By ablating such a pattern, 101 individual spectra are obtained, which were accumulated for further data processing. The integrated signals of the atomic emission lines H 656.3 nm (H-alpha), La 654.3 nm, and Li 610.4 nm were evaluated. For line integration, the software OriginPro 2016 (OriginLab Corporation, USA) was used. Signal normalization was performed using the integrated La signal, whereas differences in the La content were considered and corrected accordingly.

Calibration of the LIBS analysis was conducted by means of five ablation patterns on each calibration standard (single ablation). Since contact with air could not be avoided during preparation of the standards, potential H_2_O adsorption and/or H^+^ incorporation in the used LLZO powder has to be considered and the absolute H content of the pressed pellets is thus not directly known. To enable absolute signal quantification, the amount of additional H (*i.e.*, the H content of the used LLZO powder) was determined *via* standard addition approach using the additional H signal introduced by the added La(OH)_3_. To determine the H background originating from the instrument and to correct the analysis accordingly, the signal of the pristine (H-free) Ga:LLZO single crystal calibration standard was used.

Depth profiling experiments were conducted by ablating the same area (represented by laser pattern) 15 times in a row. Per sample one depth profiling measurement was performed. After the experiments, the sampling depths were determined using a DektakXT profilometer (Bruker, USA). A constant ablation rate was assumed in order to calculate how much material was removed with each individual ablation pattern.

### 2.4 Electrochemical impedance spectroscopy

The ionic conductivity of the LLZO samples was measured by electrochemical impedance spectroscopy (EIS) before and after the Li^+^/H^+^ exchange experiment. As electrodes, 200 nm thick Au layers were deposited on the top and bottom side of the samples using a MED 020 coating system (Bal-Tec AG, Liechtenstein). For the measurements, which were performed at room temperature (25 °C), an Alpha-A high performance frequency analyzer (Novocontrol Technologies, Germany) in the frequency range from 10 Hz or 1 kHz to 10 MHz was used.

## 3 Results and discussion

### 3.1 H_2_O analysis *via* ICP-OES analysis of aqueous solutions

After exposing the Ga:LLZO single crystal as well as the Ta:LLZO polycrystalline pellet to H_2_O at 80 °C for 80 min, the concentration of Ga, La, Li, Ta, and Zr in the used H_2_O were determined *via* ICP-OES, giving access to the amounts of cations released from the samples during the exposure. The results of the ICP-OES analysis are summarized in Table 3. Comparatively large amounts of Li^+^ were released during the immersion, for both the Ga:LLZO single crystal as well as the Ta:LLZO polycrystalline pellet (approx. 4.4 and 113 μg, respectively). Also La and Zr could be detected in the solutions, however, compared to Li the measured amounts are very small (mass ratio is at least a factor of 30 lower). Since LLZO dissolution can therefore be neglected as reason for the increased Li concentrations, the ICP-OES analysis confirms that significant Li^+^/H^+^ exchange took place during the H_2_O exposure.

Amounts of cations released into the H_2_O used for sample exposure determined *via* ICP-OES, including limit of detection (LOD) (= *x̄*_blank_ + 3 × *s*_blank_; *n* = 8) and limit of quantification (LOQ) (= *x̄*_blank_ + 6 × s_blank_, *n* = 8) of the analysis. The stated measurement uncertainties correspond to the confidence intervals of the mean values derived from replicate measurements (*α* = 0.05, *n* = 3). Comparatively large amounts of Li were released, confirming Li^+^/H^+^ exchange during the experiment

**Table:** 

Ga:LLZO single crystal								
	LOD [μg]	LOQ [μg]	Analyte mass [μg]					Analyte released from sample [%]
			0–5 min	5–30 min	30–55 min	55–80 min	Total	
Ga	0.12	0.25	<LOD	<LOD	<LOD	<LOD	—	—
La	0.07	0.11	<LOD	<LOD	<LOD	0.127 ± 0.017	0.127 ± 0.017	0.00118 ± 0.00016
Li	0.05	0.09	1.667 ± 0.006	1.349 ± 0.008	0.787 ± 0.014	0.570 ± 0.008	4.372 ± 0.019	0.3591 ± 0.0019
Ta	0.11	0.29	—	—	—	—	—	—
Zr	0.03	0.06	0.092 ± 0.007	<LOQ	<LOD	<LOQ	0.092 ± 0.007	0.00185 ± 0.00014

a Using the nominal sample composition as reference.

**Table:** 

Ta:LLZO polycrystalline pellet								
	LOD [μg]	LOQ [μg]	Analyte mass [μg]					Analyte released from sample^a^ [%]
			0–5 min	5–30 min	30–55 min	55–80 min	Total	
Ga	0.12	0.25	—	—	—	—	—	—
La	0.07	0.11	0.598 ± 0.006	0.503 ± 0.009	<LOQ	0.252 ± 0.009	1.353 ± 0.014	0.00157 ± 0.00002
Li	0.05	0.09	22.83 ± 0.05	41.39 ± 0.26	28.87 ± 0.06	19.59 ± 0.04	112.7 ± 0.27	1.226 ± 0.003
Ta	0.11	0.29	<LOD	<LOD	<LOD	<LOD	—	—
Zr	0.03	0.06	0.177 ± 0.006	0.140 ± 0.014	<LOD	<LOQ	0.316 ± 0.015	0.00119 ± 0.00008

During the immersion experiment, the water bath was replaced by fresh deionized water three times (after 5 min, 30 min, and 55 min) and all fractions were analyzed individually. Assuming that replacing the water does not substantially change the kinetics of our ion exchange, this gives access to rough time-resolved information about the occurring reaction. It can be observed that also during the last segment of the experiment a significant amount of Li^+^ was released, indicating an on-going Li^+^/H^+^ exchange even after 55 min. This is in agreement with Yow *et al.*,^22^ who shows that the ion exchange continues (at a reduced rate) up to one week when LLZO sample are immersed in water.

**Fig. 1:**
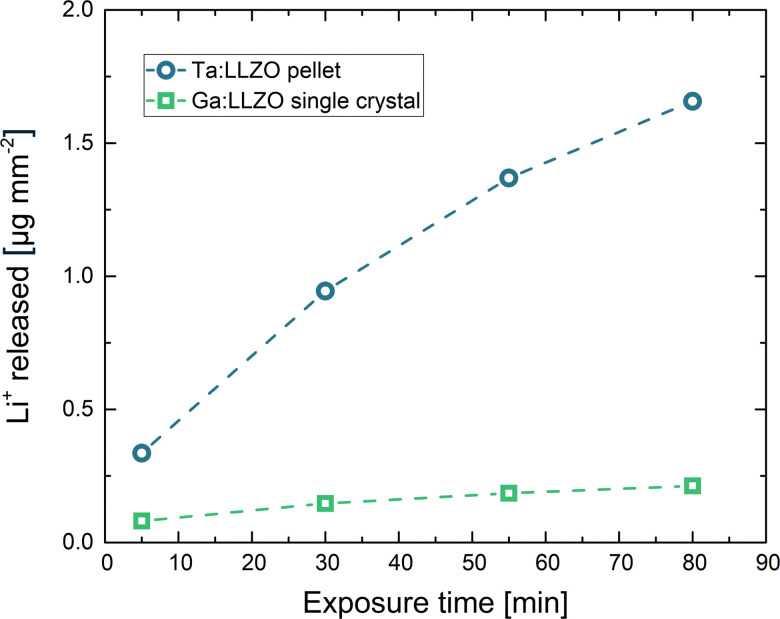
Amount of Li^+^ released during the Li^+^/H^+^ exchange experiment, normalized to the total sample surface area. Confidence intervals of the measurement (*α* = 0.05, *n* = 3) were calculated, but are too small to be visible in the plot. Significantly higher Li^+^ release for the Ta:LLZO polycrystalline pellet can be observed.

This continuous ion exchange is also visible in Fig. 1, where the total amount of released Li^+^ is plotted against the exposure time. In this plot, all values are normalized to the corresponding sample surface area (counting all sides), enabling a meaningful comparison of the two samples. It can be observed that much more Li^+^ was released from the Ta:LLZO polycrystalline pellet. At the end of the experiment, the difference to the Ga:LLZO single crystal is more than a factor of 7.5, indicating a strongly enhanced Li^+^/H^+^ exchange for the polycrystalline pellet.

If we treat the used samples as initially homogeneous semi-infinite media with surfaces being maintained at a Li^+^ concentration of zero by the water (*i.e.* assuming 100% Li^+^/H^+^ exchange at the sample surface layer), the total amount *M*^Li+^_*t*_ per surface area (in g m^−2^) of diffusing Li^+^ leaving the LLZO during the experiment can be mathematically described by^33^1
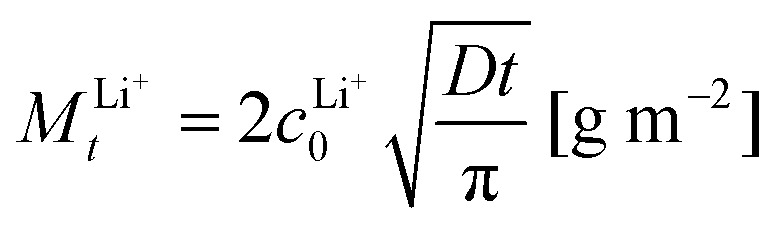
provided the diffusion distance is much smaller than the sample thickness (semi-infinite limits). Here, *c*_0_^Li^+^^ is the initial Li^+^ concentration in the sample [g m^−3^], which can easily be derived from the sample stoichiometry and the volume of a cubic LLZO unit cell (2.188 × 10^−27^ m^3^, ref. 34), *D* the diffusion coefficient [m^2^ s^−1^], and *t* the exposure time [s]. With LLZO being a fast Li-ion conductor, the Li^+^/H^+^ ion exchange is limited by the diffusion of H^+^ within the material, and *D* thus describes the H^+^ (and not the Li^+^) diffusivity. It is also important to mention that the condition of a zero surface concentration is not entirely accurate for the exchange experiment since Li^+^ accumulates in the surrounding water. However, since the Li^+^ concentration in the water stays very low, the resulting error should be minor.

**Fig. 2:**
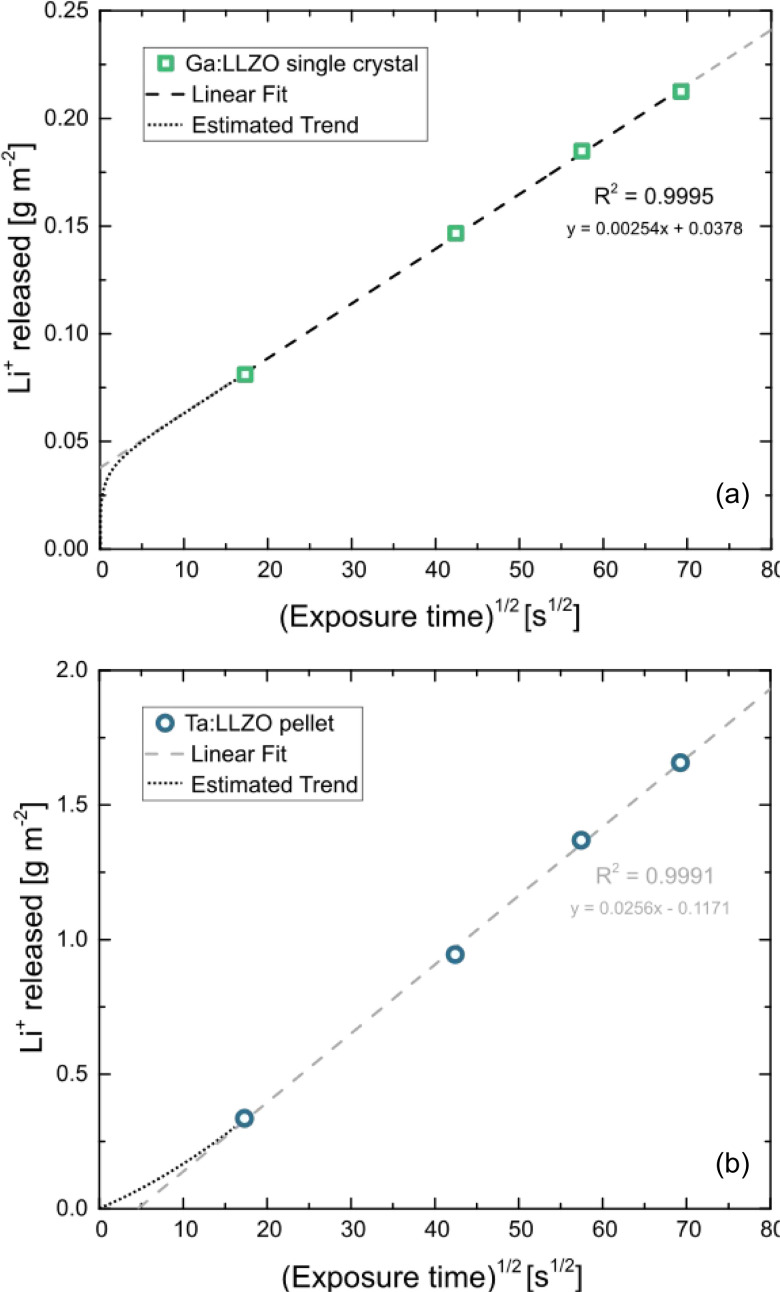
Released Li^+^*vs.* square root of the exposure time for (a) the Ga:LLZO single crystal and (b) the Ta:LLZO polycrystalline pellet. In case of the single crystal, the slope of the linear fit can be used to calculate the bulk Li^+^/H^+^ interdiffusion coefficient of LLZO.

According to eqn (1), the total amount of released Li^+^ shown in Fig. 1, which is normalized to the total sample surface and thus corresponds to *M*^Li+^_*t*_, should be directly proportional to the square root of the exposure time. To better visualize the exact relation for the LLZO samples, the corresponding plots are shown in Fig. 2. For the Ga:LLZO single crystal (Fig. 2a), indeed a distinct linear correlation between the released Li^+^ and the square root of the exposure time can be observed, confirming the diffusion driven Li^+^ release described by the model.

However, the plot shows a significant positive intercept, indicating that another Li release process is involved in the first segment of the experiment. Possibly, Li^+^ containing secondary phases (LiOH and/or Li_2_CO_3_), formed on the sample surface before the experiment due to contact with air, acted as additional Li^+^ source, despite cleaning the crystal surface by polishing directly before the analysis and minimizing contact with air (see Experimental). Since both LiOH and Li_2_CO_3_ are highly soluble, only the first water fraction is affected by their dissolution (showing an increased Li^+^ content). Accordingly, the rest of the experiment (*i.e.*, the remaining water fraction) is only affected by Li^+^/H^+^ exchange and therefore purely driven by ion diffusion. Since the Li^+^ release follows eqn (1) between minute 5 and 80, the slope of the linear fit can be used to calculate the diffusion coefficient of the Li^+^/H^+^ interdiffusion. The calculation results in a LLZO bulk diffusion coefficient *D*_bulk_ of 6.9 × 10^−17^ m^2^ s^−1^ at 80 °C, which is in good agreement with the H^+^ diffusivities determined by Brugge *et al.*^20^ (order of 10^−16^ m^2^ s^−1^ at 100 °C) and Hiebl *et al.*^15^ (approx. 2 × 10^−17^ m^2^ s^−1^ at room temperature).

At first glance also for the Ta:LLZO polycrystalline pellet a linear correlation between the released Li^+^ and the square root of the exposure time can be observed (Fig. 2b). Similar to the single crystal, the linear fit yields in an intercept differing significantly from zero, but in this case the offset is negative. The total amounts of exchanged Li^+^ are much higher than for the single crystal, which could explain that the positive *y*-axis intercept due to supposed soluble Li-containing phases is no longer visible. Nominally, the slope is almost a factor of ten larger than for Ga:LLZO single crystals and thus eqn (1) suggests a much larger (nominal) interdiffusion coefficient. However, the negative offset indicates a reduced ion diffusion rate at the beginning of the experiment, suggesting that the Li^+^/H^+^ exchange can no longer be described by the simple eqn (1). Accordingly, a (more complex) diffusion process seems to be decisive for polycrystalline LLZO, and it is thus problematic to calculate the diffusion coefficient like above. Experiments shown later in this chapter indicate a strongly enhanced H^+^ diffusion along the grain boundaries of LLZO, which explains the different diffusion behavior of the Ta:LLZO polycrystalline pellet as well as its significantly increased Li^+^ release compared to the Ga:LLZO single crystal.

### 3.2 LLZO analysis *via* LIBS

H^+^ uptake of the LLZO samples during the H_2_O exposure as well as its effect on the corresponding Li content were analyzed using LIBS. Since concentration gradients are generated during the occurring Li^+^/H^+^ exchange, depth-resolved information is necessary for a meaningful chemical analysis. Accordingly, LIBS depth profiling experiments were conducted.

#### LIBS calibration

To enable signal quantification, in-house prepared matrix-matched calibration standards with variable H content were used. To obtain such standards, calcinated Al:LLZO precursors were mixed with La(OH)_3_, acting as hydrogen source, and pressed into pellets. Additionally, a pristine and thus presumably hydrogen free Ga:LLZO single crystal was used as blank standard for background determination.

**Fig. 3:**
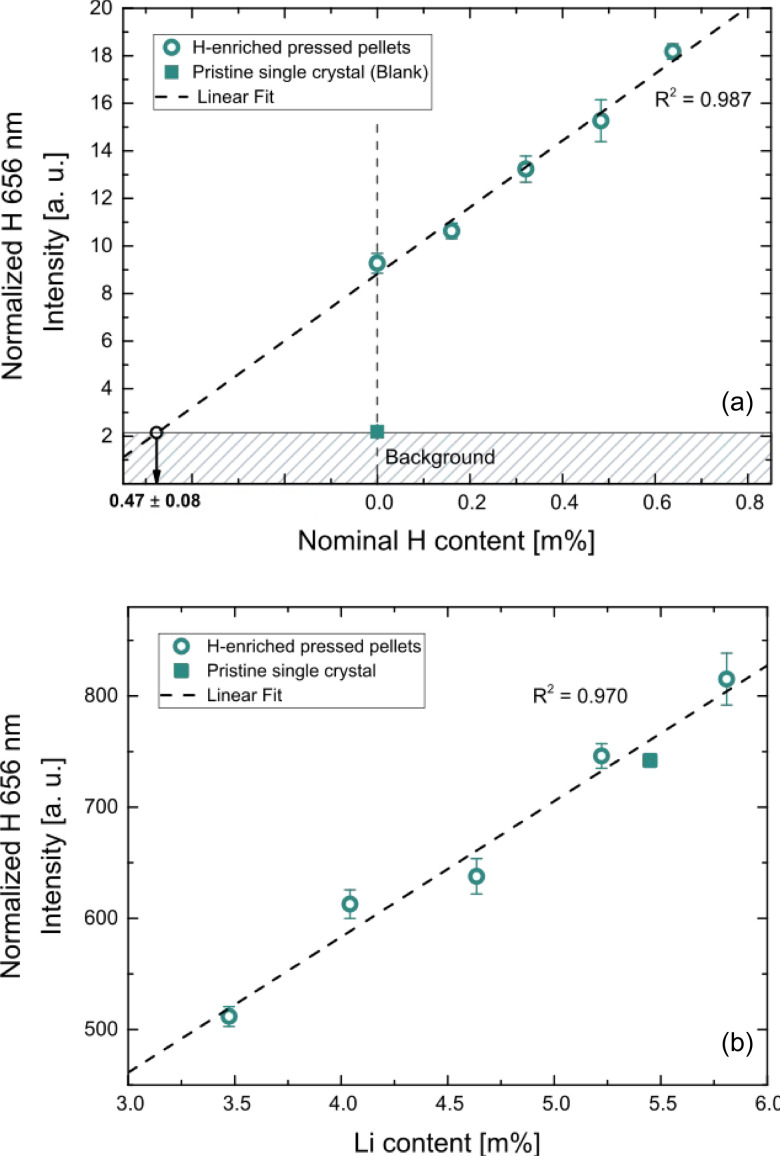
LIBS calibrations of the (a) H and (b) Li signal for different cation contents in mass %. The error bars represent the confidence intervals of the mean values derived from the measurement of multiple ablation patterns (*α* = 0.05, *n* = 5). As displayed in (a), the H content of the nominally H-free standard were determined using standard addition method. The H contents of all standards were corrected accordingly, enabling suitable signal quantification for both elements.

Prepared standards were measured using the procedure described in the experimental section, The obtained calibration curves are shown in Fig. 3. Distinct linear correlations were achieved for the H signal (Fig. 3a, *R*^2^ = 0.987) as well as the Li signal (Fig. 3b, *R*^2^ = 0.970). In Fig. 3a the nominal H content of the prepared matrix-matched standard is plotted on the *x*-axis. Since calcinated LLZO powder was used for the standard preparation and contact with air could not be avoided during the procedure, H_2_O adsorption and/or H^+^ incorporation are to be expected, affecting the actual H contents of the standards. This is confirmed by the measurement, which shows a significantly enhanced H signal. To compensate this phenomenon, the excess H was determined using the slope of the calibration curve (*i.e.*, *via* standard addition method), resulting in a H content of 0.47 m% ± 0.08 m% (95% confidence interval) for the nominally H-free standard. The H contents of the standards were corrected accordingly, enabling reliable quantification of the H signal.

#### Depth profiling

**Fig. 4:**
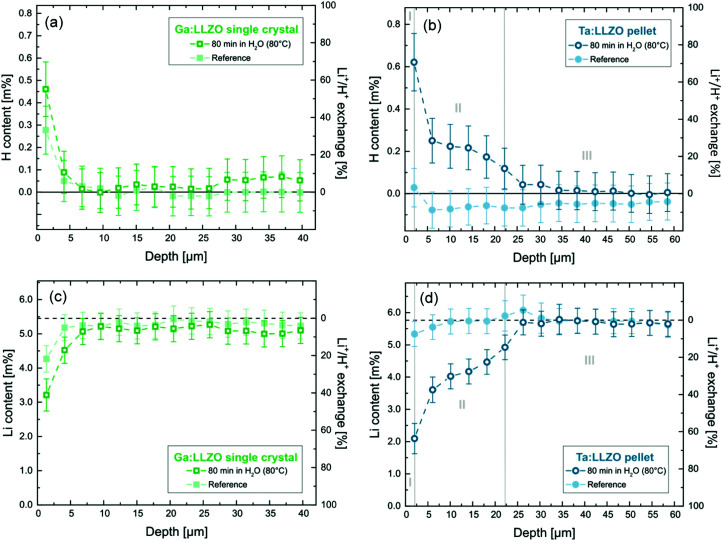
H (a and b) and Li (c and d) depth profiles (content in cation mass %) obtained by LIBS analysis of LLZO samples exposed to H_2_O as well as corresponding reference samples. Error bars represent the confidence intervals of measurements (*α* = 0.05). While for the Ta:LLZO polycrystalline pellet (b and d) up to a sampling depth of 22.2 μm significant Li^+^/H^+^ exchange can be observed, effects are much less pronounced and limited to the near surface region in case of the Ga:LLZO single crystal (a and c).

LIBS depth profiling experiments were conducted on the samples exposed to H_2_O as well as corresponding reference samples. To overcome potential differences in the ablation behavior of the investigated samples, the raw signals obtained for Li and H were normalized using the concurrently monitored La emission-line at 654.3 nm. Quantitative concentration values were derived from the recorded signal using the calibrations shown above, leading to the depth profiles displayed in Fig. 4. The measurements confirm an enhanced Li^+^/H^+^ exchange for the polycrystalline pellet, as already shown by the ICP-OES analysis (*cf.* Section 3.1), with H^+^ incorporation much deeper into the material.

Fig. 4a and b show the H depth profiles for the Ga:LLZO single crystal as well as the Ta:LLZO polycrystalline pellet, respectively. In addition to the absolute H content, the corresponding percentage of Li^+^/H^+^ exchange is shown on the secondary *y*-axis. On this scale, 100% indicates that all Li^+^ within the material was replaced with H^+^. Since in case of the Ta:LLZO pellet only the nominal sample composition is known, the average Li content of the reference sample was used as reference for the calculation. In Fig. 4c and d, the corresponding Li depth profiles are shown.

We first consider the Ga:LLZO single crystal. Here, only for the first layer (0.0–1.4 μm) a significant effect of the H_2_O exposure at 80 °C can be observed. Compared to the reference sample, the Li content is significantly lower (Fig. 4c), indicating that Li^+^/H^+^ exchange has occurred close to the surface. However, also the reference sample shows a substantial Li depletion for the first layer, even though less than the treated singly crystal. This suggests that further Li^+^/H^+^ exchange independent of the H_2_O exposure took place. Apparently, either significant ion exchange occurred during the short air exposure, or the cleaning of the sample surfaces *via* polishing before the experiments was incomplete and residues of old (H-enriched) surface layers were probed. This is in agreement with the findings of the H_2_O analysis *via* ICP-OES (*cf.* Section 3.1) indicating significant amounts of LiOH and/or Li_2_CO_3_ on the surface of the single crystal (Fig. 2a, positive intercept). Since all samples including the reference ones were dipped into cold (ultrapure) water directly before the analysis (to ensure equal measurement conditions, see Experimental), these water-soluble salts were washed away and thus did not contribute to the measured Li signal.

The H depth profile of the Ga:LLZO single crystal (Fig. 4a) is in agreement with the measured Li contents. Only for the first layer a substantially increased H concentration can be observed. Also here, sample as well as reference sample showed an effect. Absence of a statistically valid difference between the two can be explained by the lower precision of the H measurement compared to the Li analysis. Since not only the sample itself but also the sample surface is probed at the first ablation layer of the LIBS measurement, not only products of Li^+^/H^+^ exchange but also residual H_2_O adsorbates at the sample surface would lead to increased H content, potentially leading to an overestimation of the H^+^ incorporation.

In summary, the results for Ga:LLZO confirm Li^+^/H^+^ exchange caused by the H_2_O exposure at 80 °C but indicate that the effects are limited to 0.0–1.4 μm sampling depth (first layer). This is in agreement with the Li^+^/H^+^ interdiffusion coefficient determined from the measured amount of released Li^+^ (see above). From the estimate for the diffusion length *l*_D_2
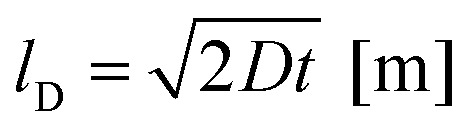
the measured bulk diffusion coefficient of 7 × 10^−17^ m^2^ s^−1^ suggests a length *l*_D_ of 0.8 μm.

Much more pronounced effects were found for the Ta:LLZO polycrystalline pellet. Fig. 4b displays the hydrogen depth profile, revealing strong differences between immersed sample and reference. The depth profile can be divided into three zones:

•Zone I (0.0–2.0 μm): Strong H^+^ incorporation (∼70% Li^+^/H^+^ exchange)

•Zone II (2.0–22.2 μm): Significant H^+^ incorporation (40–5% Li^+^/H^+^ exchange)

•Zone III (>22.2 μm): No significant H^+^ incorporation.

Zone I corresponds to the first layer of the LIBS measurement, meaning that also the sample surface might affect the analysis. However, these surface effects should be also visible for the reference sample, which does not show a significant H signal. It can thus can be assumed that here the sample surface did not significantly affect the analysis and the measured H signal mainly originates from Li^+^/H^+^ exchange. Accordingly, the measurements show that in the first 2 μm of the sample the majority of Li^+^ was replaced by H^+^. Moreover, the zone in which significant H^+^ incorporation can be observed (zone II), reaches 22.2 μm deep into the material. To the best of our knowledge, this is the highest H^+^ penetration depth after immersion in H_2_O reported in literature so far. For comparison, Brugge *et al.*^20^ found by SIMS analysis that the protonated region extends approximately 1.35 μm into a Ga:LLZO pellet after exposure at comparable conditions (100 °C, 30 min); there, however, profiles were restricted to the grain interior.

The Li depth profile of the Ta:LLZO pellet (Fig. 4d) confirms these findings. In agreement with the assumption of an interdiffusion (ion exchange), the Li content is negatively correlated to the H content and a high H content leads to a low Li content. The Li^+^/H^+^ exchange levels calculated from the determined Li contents (approx. 60% and 45–10% for zone I and II, respectively) agree well with the ones derived from the H profile, further confirming the results of the analysis and the ion exchange character. Also some electroneutral water uptake, either *via* oxygen vacancies in the bulk or at grain boundaries, cannot be excluded. However, the reasonable agreement of proton amount and amount of Li^+^ depletion suggests that other proton uptake mechanisms are of minor importance here.

To put the LIBS measurements in perspective to the H_2_O analysis *via* ICP-OES, the total amount of Li^+^ released from the Ta:LLZO pellet was calculated from the corresponding Li profile under the assumption of a uniform Li^+^/H^+^ exchange over the whole sample surface. According to this calculation, 1.39% ± 0.20% (95% confidence interval) of all Li^+^ within the specimen was released, which is in excellent agreement with the value derived from the ICP-OES analysis (1.226% ± 0.003%, *cf.*Table 3). This further supports the validity of the LIBS results and confirms the findings of this study.

**Fig. 5:**
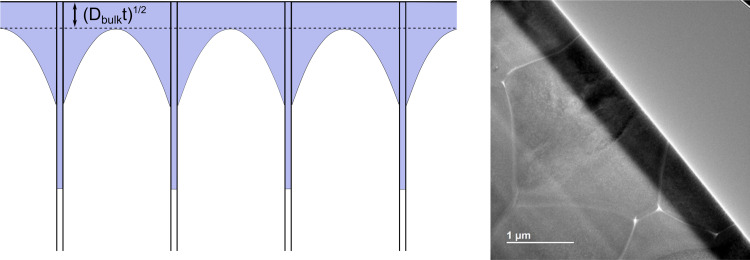
Schematic illustration of a type B diffusion kinetics according to Harrison's^35^ classification (l.h.s.) and a TEM image of the near-surface zone in a Ta:LLZO polycrystals (r.h.s.), indicating a grain size in the μm range.

The Ta:LLZO polycrystalline pellet is evidently much more prone to enhanced Li^+^/H^+^ exchange than the Ga:LLZO single crystal. This may be caused by the stabilizing element (Ta *vs.* Ga) or by the crystallinity (polycrystalline pellet *vs.* single crystal). Also in accordance with the different time dependencies in the initial phase of the ICP-OES analysis (*cf.*Fig. 2), we consider grain boundaries as the more likely reason for the enhanced ion exchange of our Ta:LLZO, Moreover, multiple studies have shown that grain boundaries are more reactive with H_2_O than grains,^6,19,20^ further supporting the hypothesis of an increased H^+^ incorporation and diffusion along the grain boundaries. At first glance, this seems to be in contradiction to the depth-resolved SIMS study by Brugge *et al.*,^20^ in which a significantly smaller H^+^ penetration depth is reported for Ga:LLZO polycrystalline pellets. However, as already mentioned above, in this experiment the probed sample area was selected in a way that only individual grains are probed, avoiding that grain boundaries contribute to the recorded signal. In the very same study, it is shown *via* EIS measurements that the grain boundary resistance increases by several orders of magnitude after immersion in H_2_O, also indicating a deeper degradation of grain boundaries compared to grains.^20^ We thus conclude that in our Ta:LLZO polycrystal hydrogen diffusion is strongly accelerated along the grain boundaries, enabling H^+^ incorporation much deeper into the material. From the (H-enriched) grain boundaries, H^+^ can then diffuse into the grains, ultimately leading to the much stronger Li^+^/H^+^ exchange for polycrystalline LLZO samples (grain structure of Ta:LLZO, see TEM image in Fig. 5, r.h.s.).

Assuming a bulk diffusion length being smaller than the grain size, this situation corresponds to the so-called Harrison type B regime of diffusion in polycrystalline materials^35,36^ (Fig. 5, l.h.s.). There, we find a depth profile with a steep zone close to the surface (related to bulk diffusion) and a shallower part which reflects grain boundary diffusion and leakage into the bulk (*cf.* model of Whipple and Le Claire^37,38^). This is exactly what we see in the measured profiles of the Ta:LLZO polycrystalline pellet (*cf.*Fig. 4b and d), and further supports the assumption of fast grain boundary diffusion. However, the data points are not sufficiently precise to deduce a reliable grain boundary diffusion coefficient from Fig. 4b.

### 3.3 Impact on the conductivity behavior

**Fig. 6:**
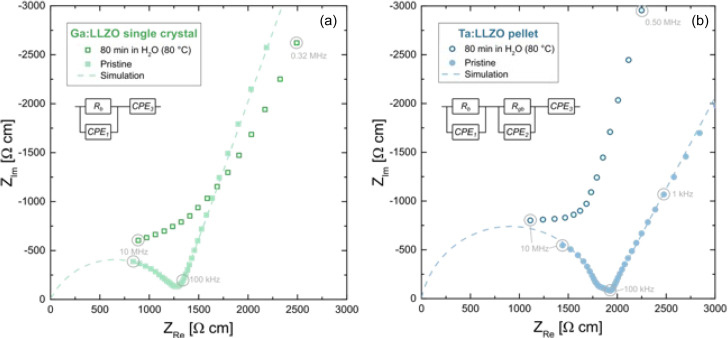
Impedance spectra of the Ga:LLZO single crystals (a) and Ta:LLZO polycrystals (b) (normalized to sample geometry). All spectra were recorded at 25 °C. Spectra of pristine samples (including fit simulation to the given equivalent circuit) are compared with samples after the exposure to water at 80 °C. Severe changes are visible.

To monitor the conductivity behavior of the LLZO samples, room temperature EIS measurements were performed before and after the H_2_O exposure. In Fig. 6, impedance spectra of the pristine (freshly polished) samples are compared with the spectra obtained after the Li^+^/H^+^ exchange experiment (80 min in H_2_O at 80 °C). The impedance spectra of both pristine LLZO samples show a part of a semicircle at high frequencies, which can be attributed to the ion conduction in the bulk, in agreement with earlier studies.^39,40^ In case of the Ta:LLZO polycrystal, a second small semicircle-like feature is visible at the intermediate frequencies, which is most likely the grain boundary contribution. At low frequencies, another contribution is observed representing the impedance of the ionically blocking Au electrodes. A fit to a simple equivalent circuit is possible for both pristine samples. The circuit consists of a R-CPE element for the bulk, a R-CPE for the grain boundary (only in case of Ta:LLZO) and a further CPE for the electrode response (CPE = constant phase element).

Bulk resistance *R*_b_ and grain boundary resistance *R*_gb_ could thus be obtained. From the total resistance *R*_total_ (= *R*_b_*+ R*_gb_, whereas *R*_gb_ = 0 in case of the Ga:LLZO single crystal), the effective ion conductivity *σ*_ion_ was calculated. For the Ga:LLZO single crystal and the Ta:LLZO polycrystalline pellet, an effective ionic conductivity of 7.8 × 10^−4^ S cm^−1^ and 5.5 × 10^−4^ S cm^−1^ was obtained at room temperature, respectively. These are typical values for cubic LLZO.^41,42^

For both the Ga:LLZO single crystal as well as the Ta:LLZO polycrystalline pellet, a severe impact of the H_2_O exposure can be observed and leads to spectra, where any clear separation into bulk, grain boundary and electrode features is lost. A strong impedance contribution in the frequency range between bulk and grain boundary is introduced, which may easily be the (inhomogeneous) interfacial zone where the ion exchange had occurred. Accordingly, also the electrical properties of the samples are strongly hampered by the Li^+^/H^+^ exchange. This also emphasizes the importance of a proper grain boundary engineering for improving the sensitivity of LLZO towards moisture during processing and under operation. However, further systematic studies (experimental as well as modeling) are needed in order to improve the understanding of the relation between ion transport along grain boundaries and the specific chemical or structural grain boundary properties. Based on this information, a rational approach to a proper grain boundary engineering may then be introduced.

## 4 Conclusions

Li^+^/H^+^ exchange of Ta:LLZO polycrystalline pellets as well as Ga:LLZO single crystals in water at 80 °C was successfully investigated by different analytical methods. From water analysis *via* ICP-OES, the time dependence of the amount of exchanged Li^+^ could be quantified. LIBS analysis of the sample, on the other hand, revealed spatially resolved information on the depletion of Li^+^ in LLZO and conversely on the depth profile of the incorporated hydrogen. These two complementary analysis approaches led to very consistent results. However, the Li^+^/H^+^ exchange behavior of the two LLZO variants is very different. In Ga:LLZO single crystal, Li^+^/H^+^ exchange is limited to the region very close to the sample surface, not extending more than 1–2 μm deep into the material. From the (small) time-dependent amounts of exchanged Li^+^ a Li^+^/H^+^ interdiffusion coefficient of 7 × 10^−17^ m^2^ s^−1^ at 80 °C could be estimated. For the Ta:LLZO polycrystalline pellet, on the other hand, pronounced Li^+^/H^+^ exchange is observed, ranging from 70% to 5% and extending as far as 20 μm deep into the sample. Most likely, a faster Li^+^/H^+^ grain boundary interdiffusion compared to the grain bulk causes the very pronounced ion exchange of polycrystalline LLZO.

## Conflicts of interest

There are no conflicts to declare.

## Supplementary Material
